# Anti-Inflammatory, Antioxidant, Metabolic and Gut Microbiota Modulation Activities of Probiotic in Cardiac Remodeling Condition: Evidence from Systematic Study and Meta-Analysis of Randomized Controlled Trials

**DOI:** 10.1007/s12602-023-10105-2

**Published:** 2023-06-22

**Authors:** Nurpudji Astuti Taslim, Muhammad Yusuf, Ade Meidian Ambari, Imke Maria Del Rosario Puling, Filzatuz Zahro Ibrahim, Hardinsyah Hardinsyah, Rudy Kurniawan, William Ben Gunawan, Nelly Mayulu, Victor F. F. Joseph, Nindy Sabrina, Mochammad Rizal, Trina Ekawati Tallei, Bonglee Kim, Apollinaire Tsopmo, Fahrul Nurkolis

**Affiliations:** 1 Clinical Nutrition, Faculty of Medicine, Hasanuddin University, Makassar, 90245 Indonesia; 2 Medical School Department, Faculty of Medicine, Brawijaya University, Malang, 65145 Indonesia; 3 Department of Cardiovascular Prevention and Rehabilitation, National Cardiovascular Center Harapan Kita, Jakarta, Indonesia; 4 Department of Cardiology and Vascular Medicine, Faculty of Medicine, Universitas Indonesia, Depok, Indonesia; 5 Division of Applied Nutrition, Department of Community Nutrition, Faculty of Human Ecology, IPB University, Bogor, West Java 16680 Indonesia; 6 Alumnus of Internal Medicine, Faculty of Medicine, University of Indonesia–Cipto Mangunkusumo Hospital, Jakarta, 10430 Indonesia; 7 Alumnus of Department of Nutrition Science, Faculty of Medicine, Diponegoro University, Semarang, 50275 Indonesia; 8 Department of Nutrition, Universitas Muhammadiyah Manado, Manado, 95249 Indonesia; 9 Department of Cardiology and Vascular Medicine, Faculty of Medicine, Sam Ratulangi University, Manado, 95115 Indonesia; 10 Nutrition Program, Faculty of Food Technology and Health, Sahid University of Jakarta, South Jakarta, Indonesia; 11 Division of Nutritional Sciences, Cornell University, Ithaca, NY 14850 USA; 12 Department of Biology, Faculty of Mathematics and Natural Sciences, Universitas Sam Ratulangi, Manado, 95115 Indonesia; 13 Department of Pathology, College of Korean Medicine, Kyung Hee University, Kyungheedae-Ro 26, Dongdaemun-Gu, Seoul, 05254 South Korea; 14 Korean Medicine-Based Drug Repositioning Cancer Research Center, College of Korean Medicine, Kyung Hee University, Seoul, 02447 Korea; 15 Department of Chemistry, Carleton University, 1125 Colonel By Drive, Ottawa, K1S5B6 Canada; 16 Biological Sciences, State Islamic University of Sunan Kalijaga, UIN Sunan Kalijaga Yogyakarta, 55281 Yogyakarta, Indonesia

**Keywords:** Cardiac remodeling, Probiotic, Heart failure, Myocardial Infarction, Gut microbiota modulation, Sarcopenia

## Abstract

Heart failure (HF) is a global pandemic with increasing prevalence and mortality rates annually. Its main cause is myocardial infarction (MI), followed by rapid cardiac remodeling. Several clinical studies have shown that probiotics can improve the quality of life and reduce cardiovascular risk factors. This systematic review and meta-analysis aimed to investigate the effectiveness of probiotics in preventing HF caused by a MI according to a prospectively registered protocol (PROSPERO: CRD42023388870). Four independent evaluators independently extracted the data using predefined extraction forms and evaluated the eligibility and accuracy of the studies. A total of six studies consisting of 366 participants were included in the systematic review. Probiotics are not significant in intervening left ventricular ejection fraction (LVEF) and high-sensitivity C-reactive protein (hs-CRP) when compared between the intervention group and the control group due to inadequate studies supporting its efficacy. Among sarcopenia indexes, hand grip strength (HGS) showed robust correlations with the Wnt biomarkers (*p* < 0.05), improved short physical performance battery (SPPB) scores were also strongly correlated with Dickkopf-related protein (Dkk)-3, followed by Dkk-1, and sterol regulatory element-binding protein 1 (SREBP-1) (*p* < 0.05). The probiotic group showed improvement in total cholesterol (*p* = 0.01) and uric acid (*p* = 0.014) compared to the baseline. Finally, probiotic supplements may be an anti-inflammatory, antioxidant, metabolic, and intestinal microbiota modulator in cardiac remodeling conditions. Probiotics have great potential to attenuate cardiac remodeling in HF or post-MI patients while also enhancing the Wnt signaling pathway which can improve sarcopenia under such conditions.

**Supplementary Information:**

The online version contains supplementary material available at 10.1007/s12602-023-10105-2.

## Introduction

Heart failure (HF) has become a global pandemic with increasing prevalence and mortality rates every year [1]. The main cause of HF is myocardial infarction (MI) which is followed by a rapid process of cardiac remodeling [2]. Besides that, disturbances in metabolic and inflammatory pathways are suspected of helping accelerate the development and progression of HF [3]. In addition, to poor prognosis HF unfortunately has a high health expenditure burden [4]. Furthermore, patients with HF experience significant functional impairment in daily activities due to muscle atrophy, weakness, and reduced endurance capacity [1, 4].

Some of the available standard management of MI treatment such as antiplatelet therapy and percutaneous coronary intervention has not been able to prevent the development of MI into HF [5]. Several breakthroughs have been made to reduce HF due to MI, such as administering angiotensin-converting enzyme (ACE) inhibitors, β-receptor blocking agents, and aldosterone receptor antagonists, but the prevalence of HF in MI patients is still high [5, 6]. In addition, this breakthrough has not been able to improve the quality of life (QoL) of MI patients [6].

Dysbiosis is a condition in which there is an alteration of the gut microbiota [7], and this condition is suspected of causing elevated levels of reactive oxygen species, lipopolysaccharides (LPSs), and harmful metabolites that can lead to cardiac hypertrophy, fibrosis, and an increase in proinflammatory cytokines, which are the primary risk factors of cardiovascular disease (CVD) [8]. According to recent investigations, probiotics offer a promising therapy to reduce inflammation, permeability, and translocation of LPSs and harmful metabolites into circulation, and oxidative stress, thereby reducing cardiac hypertrophy and contractile dysfunction in HF patients [9, 10]. In addition to reducing risk factors for cardiovascular disease, probiotics have the potential to increase patient QoL because probiotics can boost skeletal muscle mass [11].

To the best of our knowledge, no literature review regarding the effects of probiotics supplementation on attenuating cardiac remodeling following the MI is published or available. Therefore, we conducted a systematic review and meta-analysis to assess the effects of probiotics on preventing HF caused by MI.

## Materials and Methods

### Searches Strategy

Our main search for this review was randomized controlled trials (RCTs) or clinical trials that examined the impact of probiotics supplementation on reducing cardiac remodeling in the condition of HF or high risk for developing HF such as post-MI. A search for clinical trials other than RCTs was also carried out. We searched Embase, PubMed, the Cochrane Library, Wiley, and ProQuest before January 2, 2022. The search was performed using the Boolean operator method and used the following keywords: (probiotics OR synbiotics OR prebiotics) AND (cardiac remodeling OR heart failure OR cardiomyopathy OR post-myocardial infarction).

### Study Selection

#### Inclusion Criteria

1. Randomized controlled trials and clinical trials which were conducted and published within the last 10 years were used for the investigation.
2. A sample population of patients with HF fulfilling the diagnosis criteria or post-MI patients to assess the feasibility of the proposed intervention.
3. Peer-reviewed journals.
4. Studies using probiotic supplementation as its intervention.

#### Exclusion Standards

1. Patients with comorbidities or other cardiovascular disorders.
2. Patients with probiotic or standard treatment allergies.
3. Non-human trials and studies.
4. Clinical trials using a crossover study design.
5. Studies that are lacking essential outcome indices.

### Data Extraction and Outcome Measures

Four independent evaluators independently extracted the data using predefined extraction forms in a Google Sheet. Each author evaluated the eligibility and accuracy of the studies. Discussions were used to settle any disputes on the studies during the authoring phase. Each study’s data extraction yielded the following items: first author, reference, publication year, country of study, study design, sample size (male/female), age, center, participant status, dose, probiotics strain, time to follow-up, and the primary outcome (scale, baseline, post-treatment, and mean changes from baseline).

#### Main Outcome(s)

The mean deviation of biomarker values from the baseline for each trial was the subject of this review. Procollagen III, TGF-β, TMAO, matrix metallopeptidase 9 (MMP-9), and serum high-sensitivity C-reactive protein (hs-CRP) are the biomarker measures that are covered. The change in left ventricular ejection fraction (LVEF) measured by echocardiography was also analyzed as the reflection of myocardial contractility.

#### Measures of Effect

We investigated the standardized mean difference (SMD) between the probiotics intervention and the control group.

#### Additional Outcome(s)

The secondary result was observing each intervention’s adverse events and clinical symptoms.

#### Measures of Effect

The additional outcomes were reported descriptively as a percentage of both intervention and control groups.

### Risk of Bias Assessment

Using the Cochrane “Risk of Bias” 2 assessment tool [12], the quality of the included studies was assessed. This tool or software examined the following domains: randomization process, deviations from intended interventions, missing outcome data, measurement of the outcome, and selection of the reported result. The four writers carried out the process while others performed as supervisors, taking into account their prior knowledge of the tool and their experience with it. To settle any disagreements, the authors collaborated and had discussions. The domains were divided into three groups based on their level of risk of bias: low, some concern, or high risk of bias.

### Statistical Analysis

Review Manager Software (Version 5.4; Oxford, England) was used for statistical analysis. Categorical data were assessed employing odds ratio (OR) with a 95% confidence interval (CI) and continuous data were analyzed using mean difference (MD) with 95% CI. Heterogeneity among studies was assessed by the* I*-squared (*I*2) test. *I*2 ≤ 50% was considered low heterogeneity, and the fixed-effects model was adopted; otherwise, it was deemed significant heterogeneity, and the random-effects model was adopted. To avoid biases caused by methodological differences among studies, we used sensitivity analyses to find the source of heterogeneity and inconsistency. Full text was evaluated to find the research of the origins of heterogeneity and investigated its influence on meta-analysis.

### Ethical Approval

Ethical approval and informed consent of patients were not needed for the review because the authors only collected data from previous studies that had been published with their respective ethical approval.

### Protocol Register

This research scheme was conducted under the guidance of Preferred Reporting Items for Systematic Reviews and Meta-analysis (PRISMA) 2020 protocols [13], and has also been reviewed by the boards of PROSPERO-NIHR (International Prospective Register of Systematic Reviews–National Institute for Health Research) and has been registered with the number CRD42023388870.

## Results

**Fig. 1:**
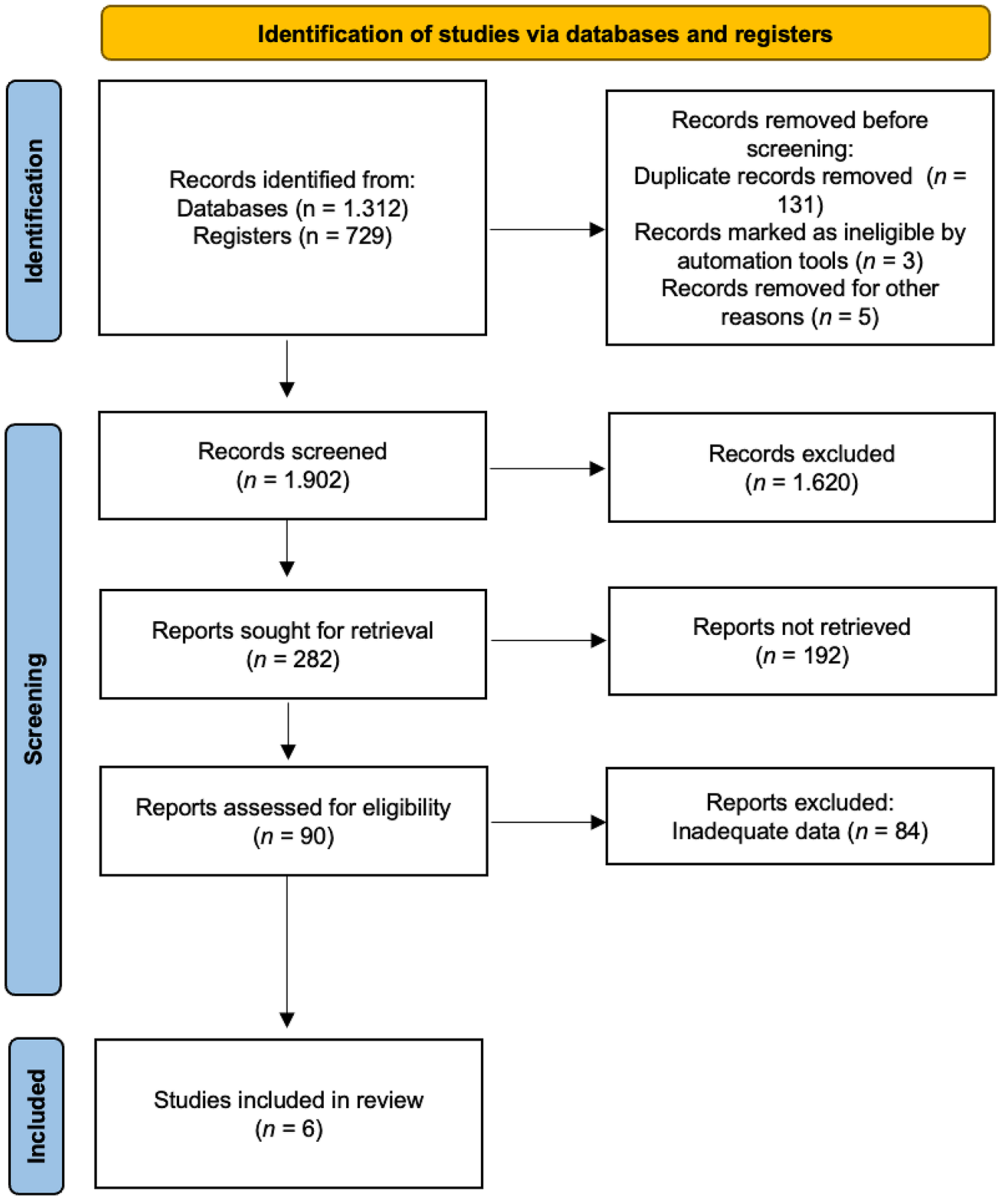
Prisma flow diagram of study selection

A total of six (6) RCTs were included in the systematic review, which yielded a total of 366 participants who were assessed using probiotics as the intervention and placebo as the control (Fig. 1).

**Fig. 2:**
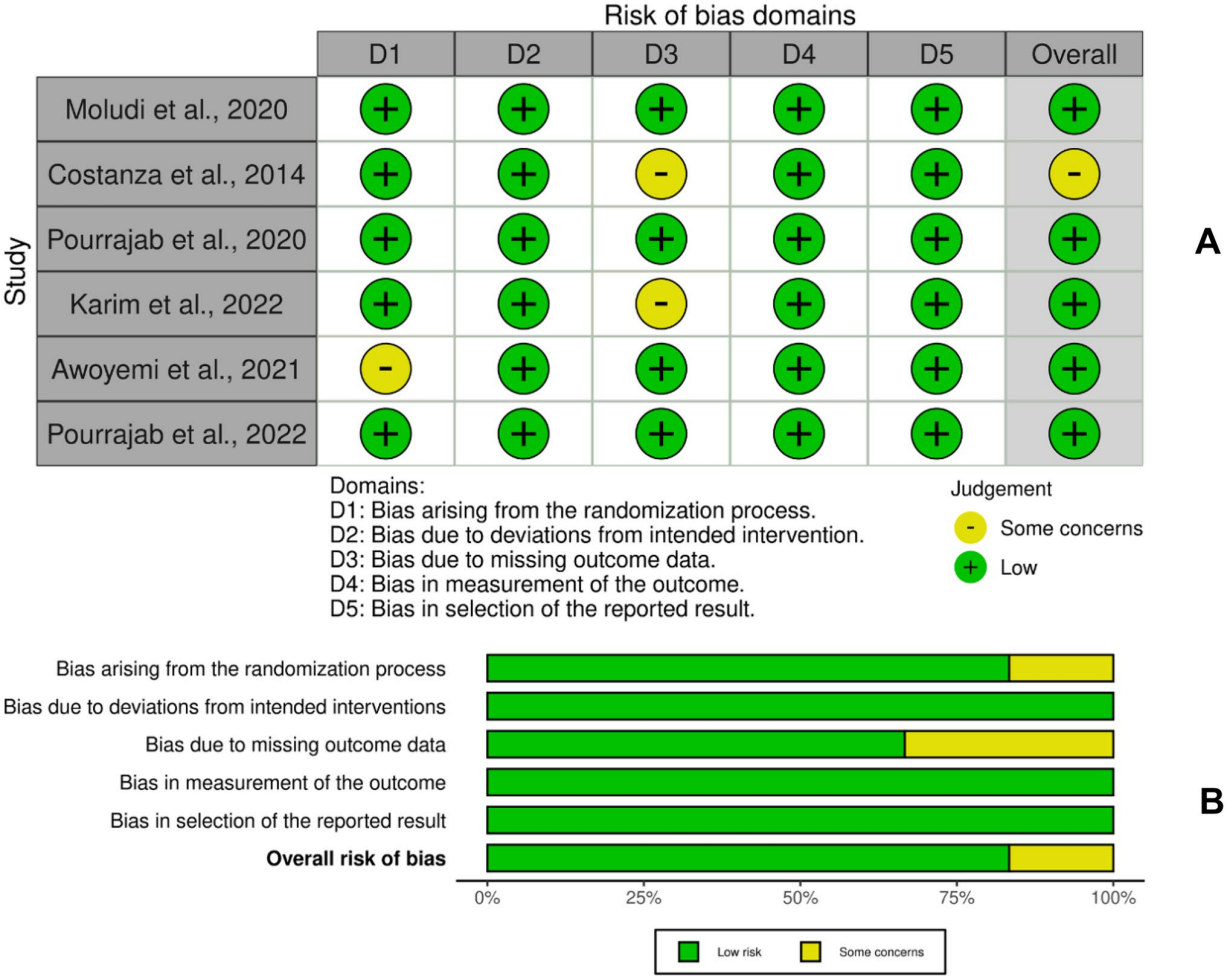
Analysis for risk of bias. Six studies were analyzed for a variety of biases using the tools in RevMan software. **A** Traffic light plot of study quality assessment based on Cochrane RoB Tool 2.0. **B** Summary plot of study quality assessment based on Cochrane RoB 2.0. The risk for bias that was analyzed was evaluated using the Revised Tool RoB 2.0, which has five domains for studies. The results were recorded in the domain file bias (.xlsx) and then processed to the ROBVIS website for display and summary of the study

Of all the included studies, three studies underwent quantitative analysis. All RCTs were also analyzed to determine the risk of bias in each study and narratively synthesize the results of each outcome reported (Fig. 2). The studies, which were published within the last 10 years, were done across various nations (Pakistan, Iran, and Brazil). The participants are post-MI or HF patients and are distributed almost equally in each gender (M/F). They were randomly assigned to an intervention group and a control group. Probiotic interventions came in a variety of probiotic strains and doses. Left ventricle ejection fraction (LVEF) and high-sensitivity C reactive protein (hs-CRP) were utilized to measure outcomes and further analyzed quantitatively. Table 1 includes a detailed list of the included studies’ characteristics.

**Table 1:** Characteristics of included studies

Author and year	Study center	Study design	Sample size (male)	Mean age	Dose	Probiotic strains	Duration of study	Primary outcomes	Secondary outcomes
Karim et al. (2022) [14]	Rehman Medical Institute, Peshawar and Teaching Hospital of Gomal Medical College, Dera Ismail Khan, Pakistan	RCT	Placebo = 48 Probiotic = 44	Placebo = 65.2 ± 5.6 Probiotic = 67.6 ± 4.9	Vivomixx 112 billion capsules once a day	Each capsule contains 112 billion live bacteria, including bifidobacteria (*Bifidobacterium longum* DSM 24736, *B. breve* DSM 24732, DSM 24737), *Lactobacilli* (DSM 24735, DSM 24730, DSM 24733, *Lactobacillus delbrueckii* subsp. *bulgaricus* DSM 24734), and *Streptococcus thermophilus* (DSM 24731)	12 weeks	Sarcopenia and functional capacity (defined by EWGSOP2): HGS, ASMI, fat mass, phase angle, SPPB	Zonulin Dkk-1 Dkk-3 SREBP1 8-isoprostanes CRP
Pourrajab et al. (2022) [15]	Shahid Rajaee Hospital and Rasoul Akram Hospital, Tehran, Iran	Triple-blind RCT	Placebo = 39 (26) Probiotic = 39 (29)	Placebo = 55.59 ± 8.95 Probiotic = 53.87 ± 7.25	Low-fat probiotic yogurt (1.5% fat) (produced by PAK Dairy Company, Tehran, Iran). Dose of 10^7^ CFU/g. The patients consumed 300 mL of yogurt daily, i.e., they received probiotics equivalent to 3 × 10^9^/day	*Lactobacillus acidophilus La5* *Bifidobacterium lactis Bb12*	10 weeks	sTWEAK	sCD163 ADMA LCAT BUN
Awoyemi et al. (2021) [16]	Multicentered (three hospitals in Norway and one hospital in Brazil)	Phase II, multicenter, open-label, RCT	Rifaximin = 48 (35) *Saccharomyces boulardii* (probiotic) = 51 (41) Placebo = 52 (39)	Rifaximin = 59 ± 10 *S. boulardii* (probiotic) = 62 ± 8 Placebo = 60 ± 10	Two capsules of 250 mg of the probiotic yeast *S. boulardii* (CNCM I-745) twice a day	*S. boulardii*	3 months	LVEF	Baseline-adjusted NT-proBNP Hs-CRP Changes in the composition of the microbiota (Shannon index, amplicon sequence variants (ASVs), and other compositional changes)
Moludi et al. (2021) [17]	Cardiology Clinic at the Shahid Madani Heart Center, Iran	Double-blind RCT	Placebo = 22 Probiotic = 22	Placebo = 56.70 ± 9.10 Probiotic = 57.10 ± 7.80	One probiotic capsule daily 1.6 × 10^9^ CFU	*Lactobacillus rhamnosus* GG	3 months	Serum procollagen III TGF-β TMAO MMP-9 hs-CRP	LVEDV LVESV LVEF
Pourrajab et al. (2020) [18]	Shahid Rajaee Hospital as well as Rasoul Akram Hospital, Tehran, Iran	Triple-blind RCT	Placebo = 39 (26) Probiotic = 39 (29)	Placebo = 55.59 ± 8.95 Probiotic = 53.87 ± 7.25	Low-fat probiotic yogurt (1.5% fat) (produced by PAK Dairy Company, Tehran, Iran). Dose of 10^7^ CFU/g. The patients consumed 300 mL of yogurt daily, i.e., they received probiotics equivalent to 3 × 10^9^/day	*Lactobacillus acidophilus La5 Bifidobacterium lactis Bb12*	10 weeks	Serum pentraxin3	NT-proBNP oxLDL ApoB100
Costanza et al. (2015) [19]	Antonio Pedro Universiy Hospital, Brazil	Triple-blind RCT	Placebo = 7 Probiotic = 7	-	1000 mg per day	*S. boulardii*	3 months	Glycemia Total cholesterol Leukocyte count Creatinine Uric acid Hs-CRP LVEF Left atrial diameter	

*EF *ejection fraction, *HFrEF *heart failure with reduced ejection fraction, *EWGSOP2 *European Working Group on Sarcopenia in Old People, *HGS *hand grip strength, *ASMI *appendicular skeletal mass index, *SPPB *short physical performance battery, *Hs-CRP *high sensitivity C-reactive protein, *TMAO *trimethylamine *N*-oxide, *MMP-9 *matrix metallopeptidase 9, *LVEF *left ventricle ejection fraction, *LVEDV *left ventricular end-diastolic volume, *LVESV *left ventricular end-systolic volume

### Left Ventricular Ejection Fraction (LVEF)

**Fig. 3:**
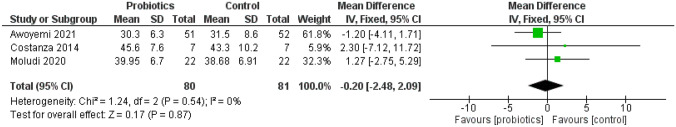
Forest plot probiotics vs. placebo regarding left ventricle ejection fraction (LVEF)

There were 3 studies that reported probiotics as an intervention vs placebo as a control with LVEF as their outcome measure (Fig. 3). The result was insignificant with *p* > 0.05 (*p* = 0.87) and MD value: − 0.20 (95% CI: − 2.48 to 2.09, *I*^2^ = 0%). However, the heterogeneity is insignificant. All things considered, it can be concluded that probiotics are not significant in intervening LVEF in the intervention group compared to the control group.

### High-Sensitivity C-Reactive Protein (hs-CRP)

**Fig. 4:**
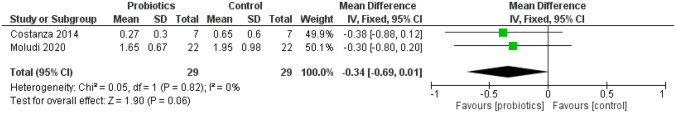
Forest plot probiotics vs. placebo regarding high-sensitivity C-reactive protein (hs-CRP)

Probiotics were reported in 2 studies as an intervention versus placebo as the control in assessing hs-CRP (Fig. 4). With *p* > 0.05 (*p* = 0.06) and an MD value of − 0.34 (95% CI: − 0.69 to 0.01, *I*^2^ = 0%), the outcome was not statistically significant though the heterogeneity is insignificant. All things considered, it can be said that probiotics had no discernible effect on hs-CRP when compared between the intervention group and the control group due to inadequate studies supporting its efficacy. However, in a single study, it reduced hs-CRP significantly compared to the placebo [17]. In the other study, it reduced hs-CRP insignificantly, while the placebo group showed a significant increase in hs-CRP [19].

### Biomarker Parameters

This systematic review included articles that reported different biomarkers. One of the biomarkers mentioned is zonulin, which serves as an indicator of intestinal permeability [14]. Other reported inflammatory biomarkers besides hs-CRP are CRP and sTWEAK [17, 19]. The reported oxidative stress biomarkers are 8-isoprostane and Ox-LDL [14]. Biomarkers in the Wnt signaling pathway which is correlated with sarcopenia were also reported in one study, including the Dkk-1, Dkk-3, and SREBP1 biomarkers [14]. In addition, various biomarkers were also reported, such as sCD163, ADMA, LCAT, BUN, pentraxin3, and ApoB100 [15]. Cardiac remodeling biomarkers include TGF-β, TMAO, MMP-9, procollagen III, and NT-proBNP (Table 2).

**Table 2:** Narrative summary of the outcomes

**Outcomes**	**Probiotic**	**Baseline/placebo**	**Comments**	**Ref**
Sarcopenia and functional capacity				
HGS	25.78 ± 3.56*	23.11 ± 3.18	Probiotics improved the phase angle, HGS, and gait speed compared to baseline (*p* < 0.05)	Karim et al. (2022) [14]
ASMI (kg/m^2^)	7.59 ± 1.26	7.27 ± 1.19		
Fat mass (%)	29.78 ± 4.4	29.19 ± 3.9		
Phase angle	5.79 ± 0.47*	5.51 ± 0.37		
Gait speed (m/s)	0.98 ± 0.19*	0.83 ± 0.14		
Sarcopenia indices	12 (27.2)	14 (31.8)		
Biomarkers				
Zonulin (ng/mL)	2.51 ± 0.33*	2.82 ± 0.35	Probiotics reduced plasma zonulin, Dkk-3, SREBP1, and 8-isoprostanes, and improved plasma Dkk-1 levels in CHF patients (*p* < 0.05). However, probiotics did not affect plasma CRP levels	Karim et al. (2022) [14]
Dkk-1 (ng/mL)	13.85 ± 2.44*	12.83 ± 2.84		
Dkk-3 (ng/μL)	8.89 ± 1.93*	10.06 ± 2.41		
SREBP1 (ng/mL)	1.51 ± 0.19*	1.76 ± 0.27		
CRP (mg/L)	2.79 ± 0.44	3.08 ± 0.51		
8-isoprostanes (pg/mL)	75.29 ± 17.4*	87.31 ± 21.44		
sTWEAK (ng/L)	691.84 (335.60, 866.95), < 0.001	581.96 (444.99, 929.40), < 0.001	The difference in biomarker level changes between the two groups was not significant in all parameters (*p* > 0.05), except in sTWEAK after adjusting for confounding variables (adjusted *P*-value: 0.038)	Pourrajab et al. (2022) [15]
sCD163 (ng/mL)	0.046 (− 1.28, 1.07), 0.446	− 0.12 (− 0.98, 1.89), 0.539		
ADMA (ng/L)	1327.00 (723.00, 1735.00), 0.003	1255.00 (740.00, 2005.00), < 0.001		
LCAT (U/L)	65.40 (56.19,79.50), < 0.001	57.00 (45.80, 71.27), < 0.001		
BUN (mg/dL)	3.12 ± 7.97, 0.019	− 0.92 ± 11.21, 0.610		
Procollagen III (mg/L)	− 1.35 (− 3.10, 1.14), 0.055	− 0.0 (− 1.03, 0.10), 0.620	Significant decreases were seen in serum TGF-β concentrations (*p* = 0.001) and TMAO levels (*p* = 0.043)	Moludi et al. (2021) [17]
TGF-β (pg/mL)	** − 8.0 (− 15, − 2.1), 0.021**	− 4.0 (− 8.70, − 1.8), 0.098		
TMAO (pg/mL)	** − 17.43 (− 35.6, − 3.55), 0.019**	− 4.54 (− 15.70, 3.80), 0.165		
MMP-9 (nmol/mL)	** − 4.01 (− 15, − 0.12), 0.049**	** − 4.1 (− 2, − 0.15), 0.001**		
Serum pentraxin3 (ng/mL)	− 0.642 (− 0.79, − 0.47), < 0.001	− 0.609 (− 0.87, − 0.46), < 0.001	No significant differences between the changes of both groups (Adjusted *P*-value = 0.512)	Pourrajab et al. (2020) [18]
NT-proBNP (pg/mL)	36 (− 41, 141), 0.049	− 9 (− 102, 192), 0.118	No significant differences between the changes in both groups (adjusted *p*-value = 0.467). The probiotic yogurt group showed a significant increase in NT-proBNP from baseline to week 10	
Ox-LDL (ng/L)	− 940.50 (− 1092.20, − 395.60), < 0.001	− 438.30 (− 1000.00, − 210.70), < 0.001	The probiotic group reduced Ox-LDL significantly more than the control group (adjusted p-value = 0.046)	
ApoB100 (mg/dL)	− 6.00 (− 19.00, 14.00), 0,380	− 12.00 (− 52.00, − 6.00), < 0.001	No significant differences between the changes in both groups (adjusted *p*-value = 0.268)	
Laboratory parameters				
Glycemia (mg/dL)	94.7 ± 3.9	100.67 ± 13.1	Probiotics improved total cholesterol (*p* = 0.01) and uric acid (*p* = 0.014) compared to baseline in HF patients	Costanza et al. (2015) [19]
Total cholesterol (mg/dL)	143.2 ± 39.7*	150.83 ± 27.3		
Leukocyte count	5783.3 ± 1616	5885 ± 1563		
Creatinine (mg/dL)	0.9 ± 0.2	1.12 ± 0.4		
Uric acid (mg/dL)	5.1 ± 1.3*	6.15 ± 1.3		
Echocardiographic parameters				
LVEDV	− 7.77 (− 15.25, 2.1), 0.135	− 5.5 (− 15.8, 4.9), 0.263	Neither between-group differences nor within-group variations reached statistical significance for both variables	Moludi et al. (2021) [17]
LVESV	− 2.81 (− 14.3, 8.1), 0.604	2.24 (− 13.1, 8.1), 0.882		
Left atrial diameter	4.2 ± 0.9*	4.49 ± 0.8	Probiotics significantly reduced left atrial diameter (*p* = 0.044)	Costanza et al. (2015) [19]
Microbiota parameter				
Butyrate-acetoacetate CoA transferase gene	Log mean difference = 0.22 (− 0.05, 0.48), *P* = 0.10		No significant difference at 3 months between the standard of care and probiotic (*S. boulardii*)	Awoyemi et al. (2021) [16]

Data are presented as mean (standard deviation) for parametric quantitative data and median (25th and 75th percentiles) for non-parametric quantitative data. Except for Costanza et al. (2015), Awoyemi et al. (2021), and Karim et al. (2022) [14, 16, 19], the data are presented as changes from the baseline to the endpoint and the respective *p*-value from each group

*HGS *hand grip strength, *ASMI *appendicular skeletal mass index, *SPPB *short physical performance battery, *TMAO *trimethylamine *N*-oxide, *MMP-9 *matrix metallopeptidase 9, *LVEDV *left ventricular end-diastolic volume, *LVESV *left ventricular end-systolic volume

* = Significant difference compared to baseline

Probiotics exhibited an improvement effect in intestinal permeability by reducing zonulin. Probiotics also have anti-inflammatory properties as seen from a decrease in CRP and sTWEAK, which leads to a decrease in oxidative stress marked by a decrease in oxidative stress markers such as 8-isoprostane and Ox-LDL. In the end, probiotics reduce several cardiac remodeling triggers, including TGF-β and TMAO [17]. Probiotics also improved Wnt signaling in HF patients, which correlated with an improvement in sarcopenia.

### Echocardiographic Parameters

Two studies reported echocardiographic parameters other than LVEF, including LVEDV, LVESV, and left atrial diameter. Probiotics appeared to improve LVEDV and LVESV but both measurements did not reach statistical significance [17]. In the other study, probiotics improved left atrial diameter by reducing significantly from baseline measurement [19].

### Sarcopenia and Functional Capacity

One study featured sarcopenia indices and functional capacity outcomes, including hand grip strength (HGS), appendicular skeletal mass index (ASMI), fat mass, phase angle, gait speed, and short physical performance battery (SPPB) (Table 2). Compared to the baseline, there was an improvement in phase angle, HGS, gait speed, and SPPB. Among sarcopenia indexes, HGS showed robust correlations with the three Wnt biomarkers (*p* < 0.05). Improved SPPB scores were also strongly correlated with Dkk-3, followed by Dkk-1, and SREBP1 (*p* < 0.05).

### Miscellaneous Laboratory and Microbiota Parameters

There is one trial that showed laboratory results in the form of blood sugar, total cholesterol, leukocyte count, creatinine, and uric acid changes after the intervention of probiotics and placebo [19]. The probiotic group showed improvement in total cholesterol (*p* = 0.01) and uric acid (*p* = 0.014) compared to the baseline, while the other parameters did not reach statistical significance in changes. Furthermore, one RCT analyzed the abundance of the butyrate-acetoacetate CoA transferase gene 3 months after intervention in the Norwegian study participants. No difference was observed in levels at 3 months between the control group and probiotic group (*S. boulardii*) (Table 2).

## Discussion

**Fig. 5:**
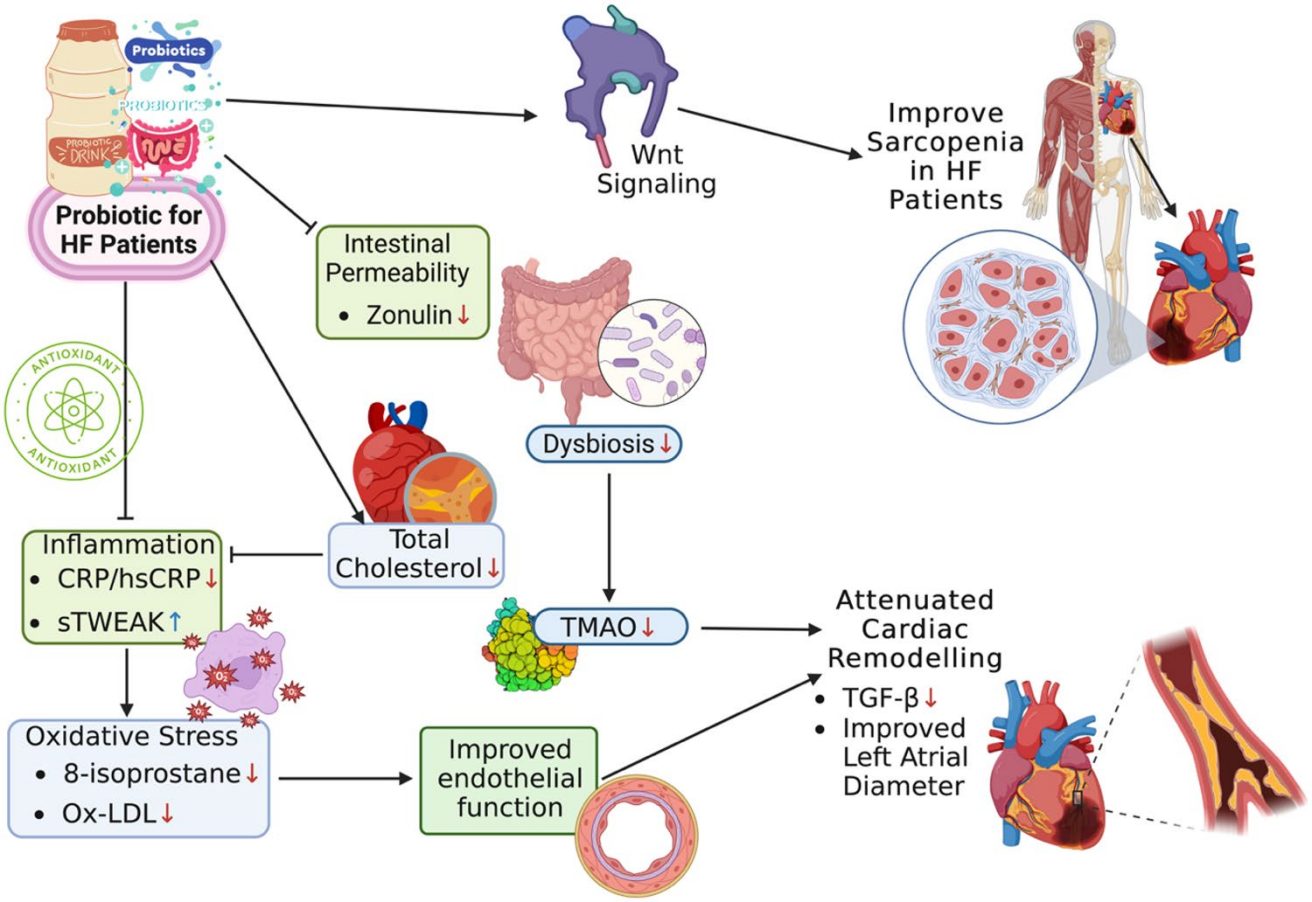
Mechanism of probiotics in cardiac remodeling condition with amelioration of sarcopenia under such conditions. Created by Fahrul Nurkolis using Biorender Premium

Numerous research studies have shown mechanisms connecting the gut microbiota’s function in low-grade inflammation in cardiovascular disease (CVD) situations in recent years [20]. Numerous investigations have also revealed a connection between the development of HF and gut microbiota [21]. Alteration in gut barrier function (dysbiosis) also leads to increased TMAO levels [22]. It has been also found that TMAO levels are substantially higher in individuals with HF compared with that in control subjects [23] and additionally, TMAO-induced cardiac hypertrophy and cardiac fibrosis [24]. Furthermore, TMAO levels are strongly associated with gut microbiota and it has been found that gut microbiota modulation using probiotics can lead to a decrease in TMAO levels [25]. Still, the precise processes through which probiotics may influence the cardiac remodeling process are unknown. TMAO, short-chain fatty acids (SCFAs), and bile acids are examples of gut microbiota metabolites that may have an impact on the development of HF [26]. Kombucha probiotic drink from butterfly pea flower was shown to modulate gut microbiota and metabolic syndrome markers paired with considerable antioxidant and metabolite compounds [27]. Metabolic endotoxemia might benefit from probiotic use (Fig. 5) [28].

Additionally, the microbiota in HF patients may have a diminished ability to synthesize advantageous metabolites such SCFAs. Maintaining the mucosal barrier of the gut requires SCFAs, particularly butyrate. Loss of barrier function may make it easier for bacteria’s lipopolysaccharides to leak out (LPS). These substances may in turn trigger pattern recognition receptors in the innate immune system. These processes could be a factor in the low-grade systemic inflammation found in HF [29–31]. Fundamentally, SCFAs were proven to contribute beneficial effects to CVD [32]. The pathology of HF may be significantly influenced by SCFAs, possibly through an agonistic effect on G-protein-coupled receptors, inhibition of histone deacetylases (HDACs), restoration of mitochondrial function, amelioration of cardiac inflammatory response, use of SCFAs as an energy source, and distant effects attributed to a protective effect on other organs. Collectively, SCFAs may be an important mediator in the gut-heart axis in the pathophysiology of HF [33]. Two additional SCFAs, acetate and propionate, may also influence the renin-angiotensin system via G-protein-coupled olfactory receptors, establishing a link between the gut microbiota and the activation of neurohormonal pathways in HF. Additionally, acetate has been demonstrated in experimental investigations to lessen cardiac hypertrophy, lessen cardiac fibrosis, and enhance cardiac function [34–36].

This is the first systematic review and meta-analysis to examine the potential of probiotics in HF and cardiac remodeling (Fig. 5). All randomized controlled trials on the mentioned topics were included and analyzed quantitatively and narratively. Comprehensiveness and novelty are the main strengths of this systematic review. Nevertheless, there are several limitations to this systematic review. First, studies regarding the effects of probiotics on cardiac remodeling are still very heterogeneous in terms of outcomes and also the study population. This problem certainly arises from the very complex nature of the course of cardiac remodeling. This has an impact on the lack of certainty of evidence presented in this systematic review. Going forward, the provision of randomized clinical trials and updating of systematic reviews is needed to increase the certainty of evidence on this topic. Second, the available studies still use different kinds and doses of probiotics between studies. This problem makes the review results ambiguous and less specific. Lastly, the number of samples included in this analysis was relatively small given the short duration of follow-up or intervention to observe cardiac remodeling.

## Conclusions

Probiotics supplementation can increase anti-inflammatory with anti-oxidant properties and accompanied by metabolic and gut modulation activities in the condition of cardiac remodeling. Therefore, it has a great potential to attenuate or prevent cardiac remodeling in HF or post-MI patients. Probiotics also enhance the Wnt signaling pathway which could improve sarcopenia under such conditions.

## Supplementary Information

Below is the link to the electronic supplementary material.

Supplementary file1 (DOCX 17 KB)

## Data Availability

No data are associated with this article.
