# Supplementary material for: Anti-Inflammatory, Antioxidant, Metabolic and Gut Microbiota Modulation Activities of Probiotic in Cardiac Remodeling Condition: Evidence from Systematic Study and Meta-Analysis of Randomized Controlled Trials
Source: Probiotics Antimicrob Proteins. 2023 Jun 22;15(4):1049–61. doi: 10.1007/s12602-023-10105-2 (PMC10393865; doi:10.1007/s12602-023-10105-2)
Supplement: Supplementary file 1 — Supplementary file1 (DOCX 17 KB) [file 12602_2023_10105_MOESM1_ESM.docx]

**Table S1**. Detailed Inclusion and Exclusion Criteria from the Included Studies

| Author & Year | Inclusion Criteria | Exclusion criteria |
| --- | --- | --- |
| Karim *et al*., 2022 | - HFrEF | - Musculoskeletal/digestive disorders - Malignancies - Prolonged bed rest in the past four weeks - Intake of probiotics/antibiotics in the past six weeks |
| Pourrajab *et al*., 2022 | - Age 30-70 - HF with EF ≤40%, NYHA I-III | - having other serious illnesses - using any nutritional supplement during the past month - using any drug or smoking - having lactose intolerance and allergy to dairy products - taking corticosteroid drugs in the last 4 weeks - receiving antibiotics in the last 3 months - having a history of gastrointestinal surgery - taking anti-inflammatories other than low-dose aspirin - having an artificial heart valve - having a history of rheumatic heart disease - pregnancy and lactation - use of insulin - BMI higher than 30 kg/m^2 |
| Awoyemi *et al*., 2021 | - HF - NYHA II-III - LVEF <40% - Optimal medical treatment for the past three months | - treatment with antibiotics or probiotics during the last three months before inclusion - signiﬁcant comorbiditie - treatment with immunosuppressive drugs - concurrent infections - bowel disease - Patients who had received cardiac resynchronization therapy during the past six months |
| Moludi *et al*., 2020 | - Patients underwent PTCA | - LVEF <35% - Failed PCI |
| Pourrajab *et al*., 2020 | - Age 30-70 - HF - LVEF ≤40% - NYHA I-III | - having other diseases (e.g., acute or chronic liver disorders, and hepatitis B and C), - diabetes, thyroid disorders, renal dysfunction (300 mmol/L ≤ creatinine and glomerular filtration rate < 30 mL/min), pulmonary diseases, known inflammatory and autoimmune diseases, cancer, rheumatoid arthritis, and acute infection - using any nutritional supplement within the past month - using drugs and smoking - having lactose intolerance and allergy to dairy products - taking corticosteroid drugs in the last 4 weeks, - receiving antibiotics in the last 3 months, - having a history of gastrointestinal surgery - taking anti-inflammatory drugs other than low doses of aspirin (80 mg/day) - having an artificial heart valve - having a history of rheumatic heart disease - pregnancy - insulin intake - Body Mass Index (BMI) >30 |
| Costanza *et al*., 2014 | - HF - NYHA II-III - LVEF <50% | - current or recent (last 4 weeks) use of corticosteroids, non-steroid anti-inﬂammatory, probiotics, or antibiotics - clinical signs or symptoms of infection, independent of location - severe life-threatening illness, inﬂammatory or autoimmune diseases, cancer, renal disease, intestinal surgery, artiﬁcial heart valve, history of rheumatic heart disease or infective endocarditis - lactose intolerance or intolerance to dairy products. |
